# Supplementary material for: Resolving the full spectrum of human genome variation using Linked-Reads
Source: Genome Res. 2019 Apr;29(4):635–45. doi: 10.1101/gr.234443.118 (PMC6442396; doi:10.1101/gr.234443.118)
Supplement: Supplemental Material [file supp_gr.234443.118_Supplemental_longranger-2.2.zip › longranger-2.2.tar.bz2!/lr2.2-public/lib/stan/cmdstan/stan/doc/api/html/singleton__argument_8hpp_source.html]

Stan: src/stan/gm/arguments/singleton\_argument.hpp Source File


|  |  |
| --- | --- |
|  | Stan  2.5.0  probability, sampling & optimization |


- Main Page
- Related Pages
- Namespaces
- Classes
- Files

- File List
- File Members

All Classes Namespaces Files Functions Variables Typedefs Enumerations Enumerator Friends Macros Pages

- src
- stan
- gm
- arguments

singleton\_argument.hpp

Go to the documentation of this file.

1 #ifndef STAN\_\_GM\_\_ARGUMENTS\_\_SINGLETON\_\_ARGUMENT\_\_BETA

2 #define STAN\_\_GM\_\_ARGUMENTS\_\_SINGLETON\_\_ARGUMENT\_\_BETA

3

4 #include <iostream>

5 #include <boost/lexical\_cast.hpp>

6 #include <stan/gm/arguments/valued\_argument.hpp>

7

8 namespace stan {

9

10  namespace gm {

11

12  template <typename T>

13  struct type\_name {

14  static std::string name() { return typeid(T).name(); }

15  };

16

17  // Specialize to something more readable

18  template <>

19  struct type\_name<int> {

20  static std::string name() { return "int"; }

21  };

22

23  template <>

24  struct type\_name<unsigned int> {

25  static std::string name() { return "unsigned int"; }

26  };

27

28  template <>

29  struct type\_name<double> {

30  static std::string name() { return "double"; }

31  };

32

33  template <>

34  struct type\_name<bool> {

35  static std::string name() { return "boolean"; }

36  };

37

38  template <>

39  struct type\_name<std::string> {

40  static std::string name() { return "string"; }

41  };

42

43  template<typename T>

44  class singleton\_argument: public valued\_argument {

45

46  public:

47

48  singleton\_argument(): \_validity("All") {

49  \_constrained = false;

50  \_name = "";

51  \_value\_type = type\_name<T>::name();

52  }

53

54  singleton\_argument(const std::string name): \_validity("All") {

55  \_name = name;

56  }

57

58

59  bool parse\_args(std::vector<std::string>& args, std::ostream\* out,

60  std::ostream\* err, bool& help\_flag) {

61  if (args.size() == 0)

62  return true;

63

64  if ( (args.back() == "help") || (args.back() == "help-all") ) {

65  print\_help(out, 0);

66  help\_flag |= true;

67  args.clear();

68  return true;

69  }

70

71  std::string name;

72  std::string value;

73  split\_arg(args.back(), name, value);

74

75  if (\_name == name) {

76

77  args.pop\_back();

78

79  T proposed\_value = boost::lexical\_cast<T>(value);

80

81  if (!set\_value(proposed\_value)) {

82

83  if (err) {

84  \*err << proposed\_value << " is not a valid value for "

85  << "\"" << \_name << "\"" << std::endl;

86  \*err << std::string(indent\_width, ' ')

87  << "Valid values:" << print\_valid() << std::endl;

88  }

89

90  args.clear();

91  return false;

92  }

93

94  }

95  return true;

96  }

97

98  virtual void probe\_args(argument\* base\_arg, std::stringstream& s) {

99

100  s << "good" << std::endl;

101  \_value = \_good\_value;

102  base\_arg->print(&s, 0, "");

103  s << std::endl;

104

105  if (\_constrained) {

106  s << "bad" << std::endl;

107  \_value = \_bad\_value;

108  base\_arg->print(&s, 0, "");

109  s << std::endl;

110  }

111

112  \_value = \_default\_value;

113

114  }

115

116  void find\_arg(std::string name,

117  std::string prefix,

118  std::vector<std::string>& valid\_paths) {

119  if (name == \_name) {

120  valid\_paths.push\_back(prefix + \_name + "=<" + \_value\_type + ">");

121  }

122  }

123

124  T value() { return \_value; }

125

126  bool set\_value(const T& value) {

127

128  if (is\_valid(value)) {

129  \_value = value;

130  return true;

131  }

132

133  return false;

134  }

135

136  std::string print\_value() {

137  return boost::lexical\_cast<std::string>(\_value);

138  }

139

140  std::string print\_valid() {

141  return " " + \_validity;

142  }

143

144  bool is\_default() {

145  return \_value == \_default\_value;

146  }

147

148

149  protected:

150

151  std::string \_validity;

152  virtual bool is\_valid(T value) { return true; }

153

154  T \_value;

155  T \_default\_value;

156

157  bool \_constrained;

158

159  T \_good\_value;

160  T \_bad\_value;

161

162  };

163

164  typedef singleton\_argument<double> real\_argument;

165  typedef singleton\_argument<int> int\_argument;

166  typedef singleton\_argument<unsigned int> u\_int\_argument;

167  typedef singleton\_argument<bool> bool\_argument;

168  typedef singleton\_argument<std::string> string\_argument;

169

170  } // gm

171

172 } // stan

173

174 #endif

stan::gm::singleton\_argument::set\_value

bool set\_value(const T &value)

**Definition:** singleton\_argument.hpp:126

stan::gm::valued\_argument::print\_help

virtual void print\_help(std::ostream \*s, const int depth, const bool recurse=false)

**Definition:** valued\_argument.hpp:26

stan::gm::singleton\_argument::\_validity

std::string \_validity

**Definition:** singleton\_argument.hpp:151

stan::gm::singleton\_argument::parse\_args

bool parse\_args(std::vector< std::string > &args, std::ostream \*out, std::ostream \*err, bool &help\_flag)

**Definition:** singleton\_argument.hpp:59

stan::gm::singleton\_argument::singleton\_argument

singleton\_argument(const std::string name)

**Definition:** singleton\_argument.hpp:54

stan::gm::type\_name< bool >::name

static std::string name()

**Definition:** singleton\_argument.hpp:35

stan::gm::singleton\_argument::\_value

T \_value

**Definition:** singleton\_argument.hpp:154

stan::gm::type\_name< int >::name

static std::string name()

**Definition:** singleton\_argument.hpp:20

stan::gm::singleton\_argument::print\_valid

std::string print\_valid()

**Definition:** singleton\_argument.hpp:140

stan::gm::singleton\_argument

**Definition:** singleton\_argument.hpp:44

stan::gm::singleton\_argument::\_bad\_value

T \_bad\_value

**Definition:** singleton\_argument.hpp:160

stan::gm::singleton\_argument::probe\_args

virtual void probe\_args(argument \*base\_arg, std::stringstream &s)

**Definition:** singleton\_argument.hpp:98

stan::gm::int\_argument

singleton\_argument< int > int\_argument

**Definition:** singleton\_argument.hpp:165

stan::gm::singleton\_argument::value

T value()

**Definition:** singleton\_argument.hpp:124

stan::gm::singleton\_argument::print\_value

std::string print\_value()

**Definition:** singleton\_argument.hpp:136

stan::gm::string\_argument

singleton\_argument< std::string > string\_argument

**Definition:** singleton\_argument.hpp:168

stan::gm::argument::split\_arg

static void split\_arg(const std::string &arg, std::string &name, std::string &value)

**Definition:** argument.hpp:54

valued\_argument.hpp

stan::gm::valued\_argument

**Definition:** valued\_argument.hpp:10

stan::gm::type\_name

**Definition:** singleton\_argument.hpp:13

stan::gm::valued\_argument::\_value\_type

std::string \_value\_type

**Definition:** valued\_argument.hpp:48

stan::gm::singleton\_argument::singleton\_argument

singleton\_argument()

**Definition:** singleton\_argument.hpp:48

stan::gm::real\_argument

singleton\_argument< double > real\_argument

**Definition:** singleton\_argument.hpp:164

stan::gm::argument::\_name

std::string \_name

**Definition:** argument.hpp:76

stan::gm::argument::indent\_width

int indent\_width

**Definition:** argument.hpp:79

stan::gm::type\_name< std::string >::name

static std::string name()

**Definition:** singleton\_argument.hpp:40

stan::gm::singleton\_argument::is\_valid

virtual bool is\_valid(T value)

**Definition:** singleton\_argument.hpp:152

stan::gm::singleton\_argument::\_constrained

bool \_constrained

**Definition:** singleton\_argument.hpp:157

stan::gm::u\_int\_argument

singleton\_argument< unsigned int > u\_int\_argument

**Definition:** singleton\_argument.hpp:166

stan::gm::singleton\_argument::is\_default

bool is\_default()

**Definition:** singleton\_argument.hpp:144

stan::gm::singleton\_argument::find\_arg

void find\_arg(std::string name, std::string prefix, std::vector< std::string > &valid\_paths)

**Definition:** singleton\_argument.hpp:116

stan::gm::argument::name

std::string name() const

**Definition:** argument.hpp:26

stan::gm::type\_name::name

static std::string name()

**Definition:** singleton\_argument.hpp:14

stan::gm::argument::print

virtual void print(std::ostream \*s, const int depth, const std::string prefix)=0

stan::gm::type\_name< double >::name

static std::string name()

**Definition:** singleton\_argument.hpp:30

stan::gm::singleton\_argument::\_default\_value

T \_default\_value

**Definition:** singleton\_argument.hpp:155

stan::gm::argument

**Definition:** argument.hpp:12

stan::gm::type\_name< unsigned int >::name

static std::string name()

**Definition:** singleton\_argument.hpp:25

stan::gm::singleton\_argument::\_good\_value

T \_good\_value

**Definition:** singleton\_argument.hpp:159

stan::gm::bool\_argument

singleton\_argument< bool > bool\_argument

**Definition:** singleton\_argument.hpp:167


---

[ Stan Home Page ]

*© 2011–2014,
Stan Development Team.*
